# Chronic Expanding Hematoma in the Liver Presenting with Intraperitoneal Hemorrhage: A Case Report and Literature Review

**DOI:** 10.70352/scrj.cr.26-0261

**Published:** 2026-06-25

**Authors:** Kosuke Funaki, Renta Kawaguchi, Takao Hara, Takashi Masuda, Yu Takeuchi, Akio Morimoto, Tsutomu Daa, Masafumi Inomata

**Affiliations:** 1 Department of Surgery, Nankai Medical Center, Saiki, Oita, Japan; 2 Department of Gastroenterological and Pediatric Surgery, Oita University, Oita, Oita, Japan; 3 Department of Diagnostic Pathology, Oita University, Oita, Oita, Japan

**Keywords:** liver, chronic expanding hematoma, hemoperitoneum, hepatectomy

## Abstract

**INTRODUCTION:**

Chronic expanding hematoma (CEH) is a rare condition in which a hematoma gradually enlarges over time after trauma or surgery. Hepatic CEH is extremely rare, and its preoperative differentiation from malignant tumors is difficult. Here, we report a case of CEH complicated by intraperitoneal hemorrhage.

**CASE PRESENTATION:**

A man in his 60s presented with epigastric pain. Contrast-enhanced CT revealed a 32-mm mass lesion in segment 2 of the liver and intraperitoneal hemorrhage with contrast extravasation adjacent to the lesion. Ruptured hepatocellular carcinoma was initially suspected, and emergency transcatheter arterial embolization achieved hemostasis. Follow-up CT demonstrated enlargement of the encapsulated hematoma near the embolized lesion. Left lateral sectionectomy was performed 33 days after embolization. Intraoperatively, a 10-cm cystic lesion with dense adhesions was identified in the left lateral section. Histopathological examination revealed a fibrous cyst wall with capillary proliferation and aggregates of histiocytes without malignant cells, leading to the diagnosis of hepatic CEH. The postoperative course was uneventful, and the patient was discharged on POD 8.

**CONCLUSIONS:**

CEH in the liver is rare, and its differentiation from malignant tumors is challenging; therefore, further accumulation of cases is required to establish optimal diagnostic and therapeutic strategies. If CEH in the liver is complicated by intraperitoneal hemorrhage, as in this case, surgical resection may need to be considered proactively.

## Abbreviations

AFP
: alpha-fetoprotein
CEH
: chronic expanding hematoma
HBV
: hepatitis B virus
HCC
: hepatocellular carcinoma
PTPE
: percutaneous transhepatic portal vein embolization
TAE
: transcatheter arterial embolization

## INTRODUCTION

CEH is a rare condition in which a hematoma that forms after trauma or surgery gradually increases in size over a long period.^1)^ It most commonly occurs in the extremities, soft tissues, or thoracic cavity, and occurrence in the liver is extremely rare.^1)^ Imaging findings often make it difficult to differentiate CEH from malignant tumors, and surgical resection is frequently required because malignancy cannot be excluded.^2–7)^ Here, we report a case of CEH that was initially diagnosed as ruptured HCC, treated emergently with endovascular therapy, and ultimately diagnosed as hepatic CEH by pathological examination after hepatectomy.

## CASE PRESENTATION

A man in his 60s presented to our hospital with epigastric pain. His medical history included bronchial asthma, pulmonary emphysema, diabetes mellitus, and hypertension. The patient had a history of HBV infection but no history of trauma.

On arrival, his temperature was 35.9°C, pulse rate 56 beats/min, blood pressure 102/66 mmHg, and oxygen saturation 100% on room air. The patient was alert. Abdominal examination revealed tenderness in the upper abdomen without guarding or rebound tenderness.

Laboratory tests showed the following values: hemoglobin, 13.9 g/dL; platelet count, 275 × 10^4^/uL; prothrombin time–international normalized ratio, 1.05; aspartate aminotransferase/alanine aminotransferase, 20/20 U/L; total bilirubin, 3.7 mg/dL; C-reactive protein, 0.59 mg/dL; AFP, 2.5 ng/mL; protein induced by vitamin K absence-II, 31.0 mAU/mL; and indocyanine green retention rate at 15 min, 9.7%.

Contrast-enhanced CT revealed a 32-mm mass lesion in liver segment 2, showing a low–high–low attenuation pattern. In addition, contrast extravasation was observed adjacent to the lesion, accompanied by intraperitoneal hemorrhage (**Fig. 1**). Based on these findings and the patient’s history of HBV infection, the initial diagnosis was intraperitoneal hemorrhage due to ruptured HCC.

**Fig. 1:**
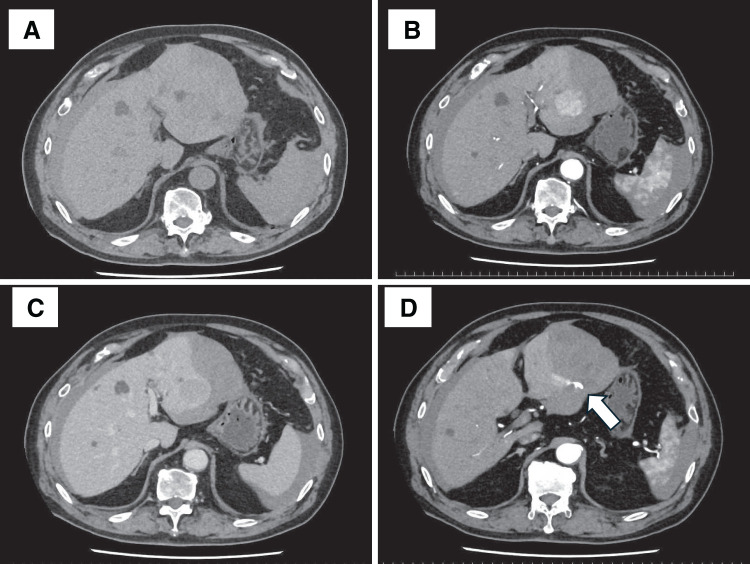
(**A**–**C**) Contrast-enhanced CT showing a 32-mm mass in segment 2 of the liver with an internal low–high–low attenuation pattern. (**D**) Contrast extravasation is seen adjacent to the lesion (white arrow).

Although the patient’s hemodynamic status was maintained, active bleeding was suspected; therefore, emergency angiography was performed. Angiography of the dorsolateral branch of the left hepatic artery revealed tumor staining and contrast extravasation caudal to the tumor. The responsible branch was embolized with gelatin sponge particles (**Fig. 2**). Post-embolization angiography confirmed the disappearance of tumor staining and extravasation.

**Fig. 2:**
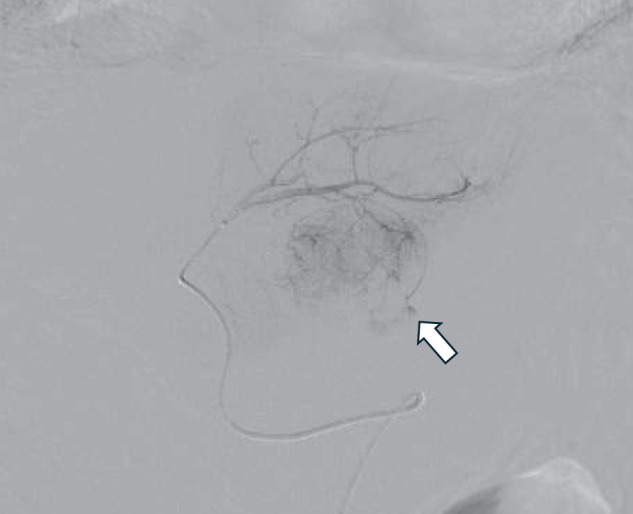
Angiography of the left hepatic arterial branch demonstrating tumor staining and contrast extravasation (white arrow). These findings disappeared after embolization.

After embolization, the patient remained hemodynamically stable without apparent rebleeding. Follow-up CT performed 1 week after the endovascular procedure showed good embolic effect; however, a 10-cm subcapsular hematoma near the mass was noted (**Fig. 3**). After stabilization of the patient’s general condition and reevaluation of the lesion, left lateral sectionectomy was performed on day 33 after embolization.

**Fig. 3:**
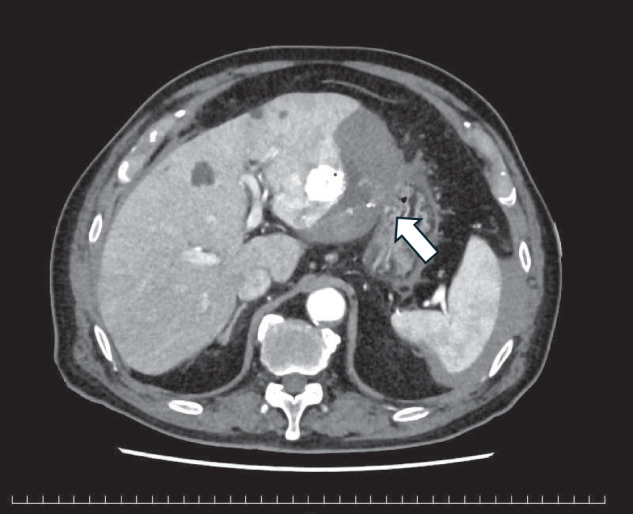
CT performed 1 week after endovascular treatment showing good retention of embolic material. Enlargement of an encapsulated hematoma adjacent to the mass is also noted (white arrow).

Because peritoneal dissemination could not be completely ruled out on preoperative imaging, exploratory laparoscopy was initially performed. The suspected lesion was found to be a hematoma attached to the greater omentum, and widespread blood adherence with dense adhesions was observed within the peritoneal cavity. Therefore, the procedure was converted to open left lateral sectionectomy to allow safe adhesiolysis and secure bleeding control. During surgery, widespread blood adherence within the peritoneal cavity was observed, and an approximately 10-cm cystic lesion was identified in the left lateral section. Dense adhesions to the surrounding tissues required time-consuming dissection and were associated with bleeding. The operative time was 436 min, and blood loss, including hematoma, was 1690 mL.

The postoperative course was uneventful, and the patient was discharged home on POD 8. During the 1-year outpatient follow-up period, there was no evidence of rebleeding or recurrence.

Histopathological examination revealed a cyst-like lesion in the resected specimen containing clot-like material (**Fig. 4**). The cyst wall comprised fibrous connective tissue with capillary proliferation and was positive for CD31 and CD34. No keratin-positive epithelial cells were identified. Aggregates of CD68-positive histiocytes were observed near the luminal surface. These findings were consistent with chronic hematoma, and no atypical cells suggestive of malignancy were present. A final diagnosis of hepatic CEH was made.

**Fig. 4:**
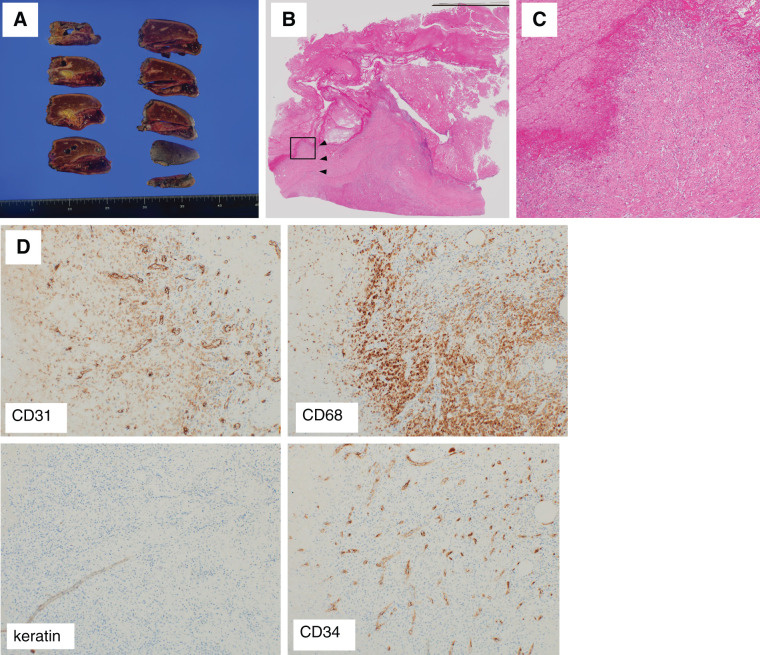
Gross and histopathological findings. (**A**, **B**) Macroscopic and loupe images show the cystic lesion (black arrowheads). (**C**) High-magnification histological image of the cyst lesion. The cyst wall was composed of clot-like material and fibrous tissue. (**D**) The lesion is positive for CD31 and CD34; the cyst-lining epithelium is keratin-negative; and CD68-positive findings suggest organized neovascularization and post-hemorrhagic changes. No atypical cells are present in the specimen.

## DISCUSSION

Our PubMed search using the keywords “chronic expanding hematoma,” “liver,” and “hepatic” (1968 to 2025) did not identify any reports of hepatic CEH presenting with intraperitoneal hemorrhage; therefore, the present case may represent the first such case reported in the literature.

CEH is a rare lesion in which an initial hematoma fails to resolve spontaneously and gradually enlarges, forming a mass that persists for more than 1 month.^1)^ Since it was first reported in 1968,^8)^ CEH has been reported predominantly in soft tissues such as the thoracic cavity, extremities, and retroperitoneum. Although trauma, surgery, and antithrombotic therapy may contribute to its development, many patients have no apparent trigger.^1)^ Pathologically, CEH is characterized by a fibrous capsule containing a mixture of old and relatively fresh hematoma components with macrophage infiltration, hemosiderin deposition, and chronic inflammatory changes. The proposed mechanisms of progression include persistent microbleeding within the fibrous capsule and ongoing rebleeding driven by activation of the fibrinolytic system. Although CEH demonstrates tumor-like enlargement, it is not a neoplastic lesion. Moreover, although a few cases of CEH rupture with hemorrhage have been reported in the skin and thoracic cavity, such events are rare, and CEH more commonly presents with compressive symptoms due to gradual enlargement.^9,10)^

Hepatic CEH is extremely rare. Including the present case, 6 cases of hepatic CEH have been identified in the literature.^2–6)^ Previously reported cases are summarized in **Table 1**. These lesions are often relatively large and tend to have a cystic architecture. CEH is commonly associated with trauma or inflammation, and some previously reported hepatic cases have occurred after a history of trauma. In contrast, in the present case, there was no history of trauma, and chronic inflammation related to prior HBV infection may have been a contributing factor, although further case accumulation is needed to clarify the underlying etiology.

**Table 1:** Previously reported cases of primary CEH in the liver

Author	Year	Age (years)	Sex	Location	Size	Treatment	Background liver
Ono et al.^2)^	2021	56	Male	Right lobe S7 (subcapsular)	17.5 × 9.5 cm	TAE, extended right sectionectomy	Not described
Taniguchi et al.^3)^	2022	57	Female	Right lobe (subcapsular)	~15 cm	Right hepatectomy	History of trauma (rib fractures)
Abe et al.^4)^	2023	73	Female	Right lobe (within a hepatic cyst)	Entire cyst: >10 cm; nodule: 25‒>35 mm	Right hepatectomy	Non-viral; details not described
Shimagaki et al.^6)^	2024	85	Male	S7 (extrahepatic protrusion)	7 cm	Partial S7 resection + cholecystectomy	No remarkable findings
Nakagawa et al.^5)^	2024	68	Male	Left lobe	~12 cm	Left trisectionectomy, PTPE, biliary drainage	Hepatic dysfunction; details unknown
Present case	2025	60s	Male	Left lobe S2 (subcapsular)	3.2 cm (bleeding cavity ~10 cm)	Emergency TAE, left lateral sectionectomy	Past HBV infection

CEH, chronic expanding hematoma; HBV, hepatitis B virus; PTPE, percutaneous transhepatic portal vein embolization; TAE, transcatheter arterial embolization

All previously reported hepatic CEH cases were from Japanese centers; however, CEH in other anatomical sites has been reported from various regions. Thus, there is currently no clear evidence of racial or ethnic predisposition, and further case accumulation is required.

The present case is distinctive in that hepatic CEH was complicated by intraperitoneal hemorrhage, resulting in a potentially life-threatening abdominal emergency requiring emergency TAE. Although the main lesion was smaller than those in previously reported hepatic CEH cases, the relationship between lesion size and hemorrhagic risk remains unclear because of the limited number of cases. Nevertheless, this case suggests that hepatic CEH may cause potentially life-threatening hemorrhage even when the lesion itself is not large.

Differentiation from malignancy based on imaging findings is difficult in hepatic cases, and surgical resection is often performed because malignant disease cannot be excluded.^2–7)^ On CT, CEH often appears as a well-marginated, low-attenuation lesion with internal heterogeneity and may be associated with calcification.^11–13)^ On MRI, high signal intensity on T1-weighted images and a mosaic pattern of low-to-high signal intensity on T2-weighted images have been described, although these findings are not highly specific.^14)^ Differential diagnoses include HCC and cystic hepatic tumors such as biliary cystadenocarcinoma. Although early enhancement and washout on contrast-enhanced CT or MRI, tumor markers, and underlying liver disease may help differentiate HCC, CEH can sometimes show similar findings, making preoperative diagnosis challenging. Intracystic nodules and elevated tumor markers may be helpful in differentiating cystic tumors; however, CEH can also show elevated tumor markers associated with inflammation or hemorrhage, limiting their utility.^15,16)^ Therefore, a comprehensive evaluation of imaging findings, laboratory data, and the clinical course is required, and a definitive diagnosis relies on histopathology.

As imaging alone cannot reliably exclude malignancy, surgical resection is often selected as both a diagnostic and therapeutic approach for CEH of the liver.^2–6,17)^

CEH may be associated with a fibrous capsule and dense adhesions, necessitating careful preoperative assessment and meticulous preparation to control bleeding.^18)^ In the present case, the lesion was initially suspected to be ruptured HCC. For ruptured HCC, staged hepatectomy after initial hemostasis by TAE has been reported as a treatment strategy, and hepatectomy is often performed 14–42 days after endovascular treatment once the patient’s condition has stabilized.^19,20)^ Therefore, in the present case, elective hepatectomy was performed 33 days after embolization. However, even at this timing, widespread blood adherence and dense adhesions were observed intraoperatively, requiring time-consuming adhesiolysis and resulting in prolonged operative time and substantial blood loss.

In addition, aspiration or drainage alone may be insufficient because incomplete evacuation can leave the capsule behind, with a risk of persistence or re-expansion. Given the potential risk of intraperitoneal hemorrhage, as demonstrated in the present case, resection may warrant consideration once CEH occurring in the liver is diagnosed or strongly suspected.

## CONCLUSIONS

Hepatic CEH is an extremely rare and benign condition that can mimic malignant liver tumors. This case suggests that patients may also present with intraperitoneal hemorrhage requiring emergency TAE. Because preoperative differentiation from malignancy remains difficult, hepatic resection may serve both diagnostic and therapeutic purposes when bleeding risk or diagnostic uncertainty is substantial.

## DECLARATIONS

### Availability of data and materials

The data are not available for public access because of patient privacy concerns, but are available from the corresponding author upon reasonable request.
